# *Pediococcus pentosaceus*-Fermented *Cordyceps militaris* Inhibits Inflammatory Reactions and Alleviates Contact Dermatitis

**DOI:** 10.3390/ijms19113504

**Published:** 2018-11-07

**Authors:** Ha-Kyoung Kwon, Min-Jung Song, Hye-Ji Lee, Tae-Sik Park, Moon Il Kim, Hye-Jin Park

**Affiliations:** 1 Department of Food Science and Biotechnology, College of BioNano Technology, Gachon University, Gyeonggi-do 13120, Korea; kyoungkh93@naver.com (H.-K.K.); manda1002@naver.com (H.-J.L.); 2 Department of, College of Food Biotechnology, Division of Bioindustry, Silla University, Busan 46958, Korea; songmj@silla.ac.kr; 3 Department of Life Science, College of BioNano Technology, Gachon University, Gyeonggi-do 13120, Korea; tspark@gachon.ac.kr; 4 Department of BioNano Technology, College of BioNano Technology, Gachon University, Gyeonggi-do 13120, Korea; moonil@gachon.ac.kr

**Keywords:** GRC-ON89A, macrophage, anti-inflammatory, DNFB-induced ACD mice, NF-κB

## Abstract

*Cordyceps militaris* is a medicinal mushroom used to treat immune-related diseases in East Asia. We investigated the anti-inflammatory effect of the extract of *C. militaris* grown on germinated *Rhynchosia nulubilis* (GRC) fermented with *Pediococcus pentosaceus* ON89A isolated from onion (GRC-ON89A) in vivo as well as in vitro. The anti-inflammatory effect of GRC-ON89A was investigated in lipopolysaccharide (LPS)-stimulated RAW 264.7 macrophages. The total polyphenol content (TPC) and total flavonoid content (TFC) in the GRC-ON89A ethanol extract were significantly increased compared to that in GRC. GRC-ON89A hexane fraction (GRC-ON89A-Hex) inhibited the release of nitric oxide (NO) compared to that of the LPS-treated control without cytotoxicity in LPS-stimulated RAW 264.7 macrophages. GRC-ON89A-Hex decreased the inducible NO synthase (iNOS), cyclooxygenase 2 (COX2), and tumor necrosis factor (TNF)-α mRNA expression in LPS-stimulated RAW 264.7 macrophages. In addition, pre-treatment with GRC-ON89A-Hex significantly inhibited LPS-stimulated phosphorylation of mitogen-activated protein kinases (MAPKs) and nuclear factor (NF)-κB. To induce allergic contact dermatitis (ACD), 1-fluoro-2, 4-dinitrofluorobenzene (DNFB) was applied to the surface of the right ears of C57BL/6N mice. GRC-ON89A reduced the ear swelling and thickness in DNFB-induced ACD mice. This study demonstrates the potential usefulness of GRC-ON89A as an anti-inflammatory dietary supplement or drug.

## 1. Introduction

Allergic contact dermatitis (ACD), a chronic inflammatory skin disease, is a type IV delayed-hypersensitivity response induced by sensitization to allergens that causes redness, edema, and dryness of the skin [1,2]. Furthermore, the prevalence of ACD in children and adults worldwide is 15–20% and 1–3%, respectively [3,4,5]. The prevalence rate of ACD is higher in young children under 10 years than it is adults [3,6]. Systemic corticosteroid or steroids combined with methotrexate, and azathioprine have been used for ACD [7]. However, long-term use of these drugs can cause unwanted side effects such as diabetes, hypertension, myelosuppression, infections, and ulcer [7]. Therefore, there is a need to develop novel agents, preferably from natural products, to treat ACD [3].

*Cordyceps militaris*, which belongs to the class Ascomycetesis, is used as a traditional medicine in East Asia due to its biological activities such as immune boosting activity. *C. militaris* extracts possess immuno-enhancing, anti-inflammatory [8,9], and anticancer activities [10]. However, to isolate the principles that exert these biological effects, an expensive extraction procedure is needed. To resolve this issue, we fermented *C. militaris* grown on germinated *Rhynchosia nulubilis* (GRC) with various lactic acid bacteria strains. Among them, *Pediococcus pentosaceus* ON89A isolated from onion (GRC-ON89A) was the most antioxidative in the 2,2-diphenyl-1-picrylhydrazyl (DPPH) photometric assay. Lactic acid bacteria have been reported to reduce allergic inflammation [11,12]. Previous studies have also reported that extracts of *C. militaris* grown on germinated soybean alleviated ACD symptoms [13].

Moreover, in our recently published paper, we reported that GRC-ON89A enhanced immune activity and contained higher levels of β-glucan, cordycepin, and short chain fatty acids (SCFAs) than GRC [14]. SCFAs are known to reduce pro-inflammatory cytokine production [15]. Administration of acetate, an SCFA, suppressed the increase in ear thickness induced by 1-fluoro-2,4-dinitrofluorobenzene (DNFB) [16]. However, the anti-inflammatory efficacy of GRC-ON89A has not yet been elucidated and, therefore, we investigated this phenomenon in lipopolysaccharide (LPS)-stimulated RAW 264.7 macrophages and in DNFB-induced ACD murine model.

## 2. Results

### 2.1. Adenosine and Cordycepin Contents in GRC-ON89A

Adenosine and cordycepin are major bioactive components in *C. militaris* and their anti-inflammatory activity have been studied [17]. We quantified the levels of adenosine and cordycepin in GRC and GRC-ON89A extract using gas chromatography-time-of-flight (GC-TOF) mass spectrometry (MS, Table 1). The level of adenosine in GRC-ON89A was higher than those in GRC (7.03 ± 0.15 mg/g vs. 3.88 ± 0.08 mg/g, respectively). The level of cordycepin in GRC-ON89A was also higher than that in GRC (1053.33 ± 11.81 μg/g vs. 180.58 ± 1.54 μg/g, respectively). These results suggest that fermentation of GRC by *P. pentosaceus* ON89A affected the contents of representative molecules.

### 2.2. Total Polyphenol Contents (TPC) and Total Flavonoid Contents (TFC) in GRC-ON89A

The antioxidant activity, TPC, and TFC of GRC and GRC-ON89A were investigated using *vitro* assays. TPC and TFC were determined using the Folin-Ciocalteu assay and aluminum chloride colorimetric method, respectively. The TPC and TFC in GRC-ON89A were 7.47 ± 0.27 mg gallic acid equivalent (GAE)/g dry mass and 15.92 ± 1.20 mg quercetin equivalent (QE)/g dry mass, respectively, and the corresponding values for GRC were 5.37 ± 0.48 mg GAE/g and 7.65 ± 1.70 mg QE/g, respectively (Table 2). The TPC and TFC in GRC-ON89A were higher than they were in GRC. Our data showed that polyphenol and flavonoid levels of GRC increased after fermentation with the probiotic strain ON89A.

### 2.3. GRC-ON89A Downregulates Nitric Oxide (NO) Secretion and Inducible NO Synthase (iNOS) mRNA and Protein Expression in LPS-Stimulated RAW 264.7 Macrophages

**Figure 1:**
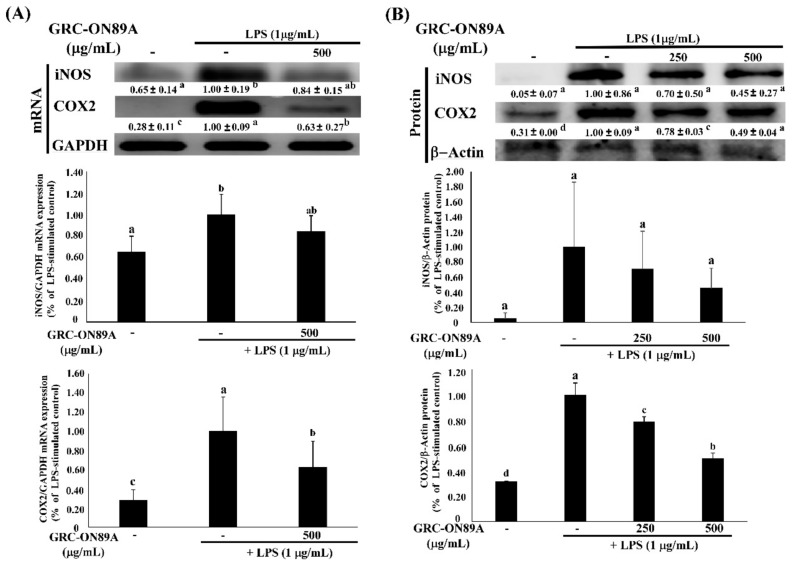
Hexane fraction of *Cordyceps militaris* grown on germinated *Rhynchosia nulubilis* (GRC) fermented with *Pediococcus pentosaceus* ON89A isolated from onion (GRC-ON89A-Hex) inhibited inflammatory cytokines. RAW 264.7 macrophages were pre-treated with GRC-ON89A-Hex extract for 2 h, and then stimulated with lipopolysaccharide (LPS, 1 µg/mL). (**A**) The levels of inducible nitric oxide synthase (iNOS), and cyclooxygenase 2 (COX2) mRNA expression were analyzed by reverse transcription-polymerase chain reaction (RT-PCR). Glyceraldehyde 3-phosphate dehydrogenase (GAPDH) was used as an internal control; (**B**) The levels of iNOS and COX-2 protein expression was measured using western blotting. β-Actin expression was used as an internal control for western blot analysis. One-way analysis of variance (ANOVA) was used to compare group means, followed by Duncan *t-*test. Different letters show significant differences between groups at *p* < 0.05. Data are expressed as means ± standard deviation (SD) of independent experiments (*n* ≥ 3).

**Figure 2:**
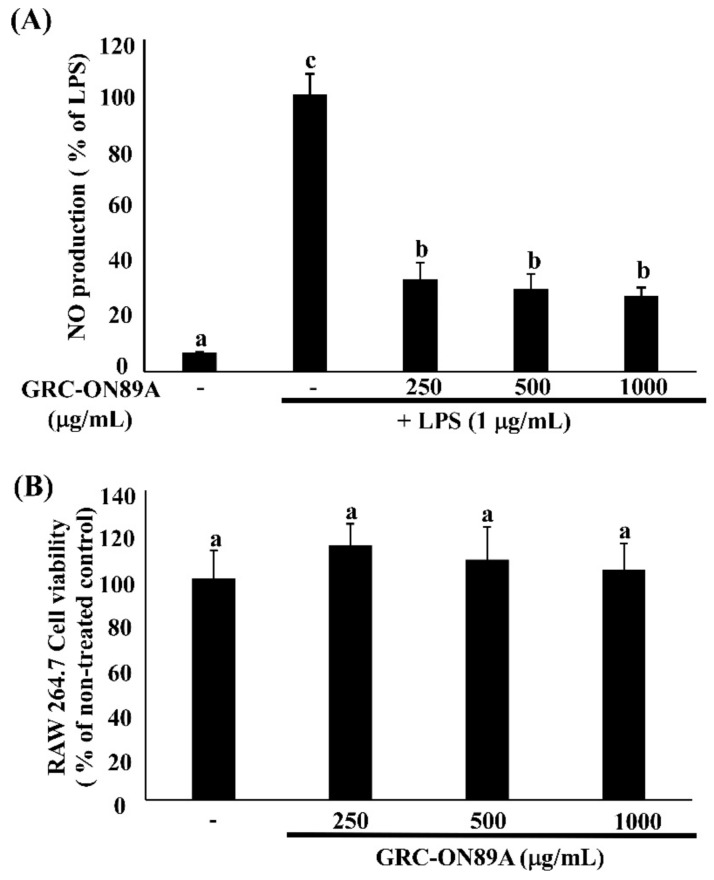
Inhibitory effects of GRC-ON89A- Hex on nitric oxide (NO) production in LPS-activated RAW 264.7 macrophages. (**A**) NO production in LPS-stimulated RAW 264.7 macrophages treated with GRC-ON89A-Hex (250, 500, and 1000 μg/mL); (**B**) Viability of RAW 264.7 macrophages after GRC-ON89A-Hex treatment (250, 500, and 1000 μg/mL). One-way ANOVA was used to compare group means, followed by Duncan *t*-test. Different letters show significant differences between groups at *p* < 0.05 (*n* ≥ 3). Data are expressed as means ± SD of independent experiments (*n* ≥ 3).

To analyze the potential anti-inflammatory properties of GRC-ON89A, we evaluated the level of inducible NO synthase (iNOS) and cyclooxygenase 2 (COX2) mRNA and protein expression in GRC-ON89A-Hex-treated RAW 264.7 macrophages using reverse transcription-polymerase chain reaction (RT-PCR) and western blot analysis. GRC-ON89A-Hex reduced NO production in LPS-stimulated RAW 264.7 macrophages. The overexpressed iNOS mRNA level was decreased slightly in LPS-stimulated RAW 264.7 macrophages following treatment with GRC-ON89A-Hex (Figure 1). In addition, the iNOS protein expression was diminished after GRC-ON89A-Hex treatment (Figure 1). Pre-treatment with 500 µg/mL GRC-ON89A-Hex in the LPS-stimulated RAW 264.7 macrophages resulted in decreased mRNA levels of COX-2 (Figure 1). Furthermore, the COX-2 protein expression was lower in GRC-ON89A-Hex treated macrophages than it was in the LPS-stimulated RAW 264.7 macrophages (Figure 1). Treatment with different concentrations of GRC-ON89A-Hex (0, 250, 500, and 1000 µg/mL) did not change the cell viability (Figure 2).

### 2.4. GRC-ON89A-Hex Inhibits mRNA and Protein Expression of Inflammatory Mediators

**Figure 3:**
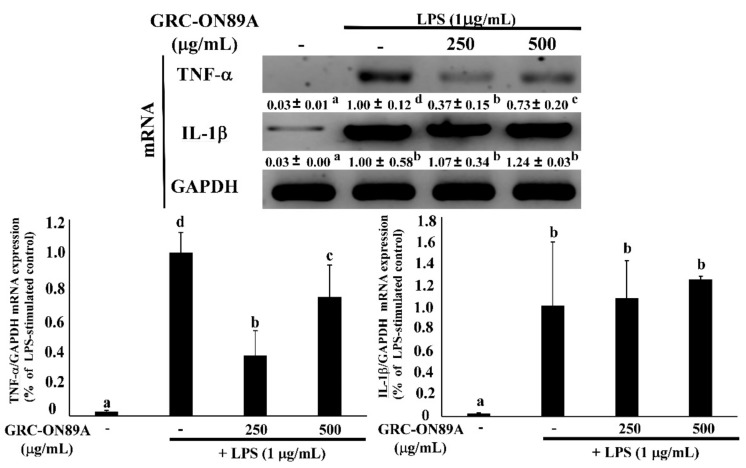
Effects of GRC-ON89A-Hex on TNF-α and IL-1β mRNA expression in LPS-stimulated RAW 264.7 macrophages. One-way analysis of variance (ANOVA) was used for comparison of group means, followed by Duncan *t*-test. Different letters indicated that the differences between the groups are significant at *p* < 0.05. Data are expressed as means ± SD of independent experiments (*n* ≥ 3).

LPS-stimulation led to overexpression of mRNA and protein of the inflammatory mediators such as interleukin (IL)-1β, and tumor necrosis factor-α (TNF-α). LPS stimulated IL-1β and TNF-α production in activated macrophages [18]. We tested whether GRC-ON89A-Hex affected the production of various inflammatory mediators using RAW 264.7 macrophages. The level of TNF-α mRNA expression significantly decreased following treatment with 250 and 500 μg/mL GRC-ON89A Hex (Figure 3).

### 2.5. GRC-ON89A-Hex Suppresses Phosphorylation of Mitogen-Activated Protein Kinases (MAPKs) and Nuclear Factor (NF)-κB p65 in LPS-Stimulated RAW 264.7 Macrophages

**Figure 4:**
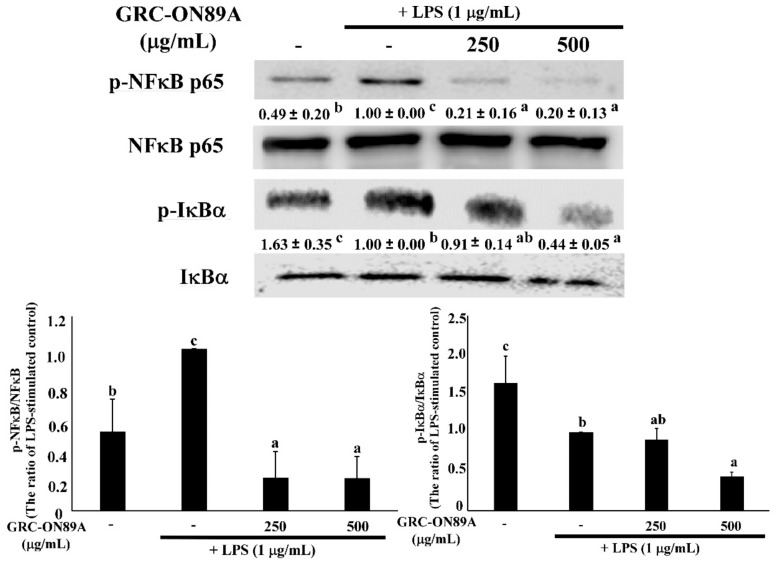
GRC-ON89A-Hex suppresses the phosphorylation of nuclear factor (NF)-κB and IκBα in LPS-stimulated RAW 264.7 macrophages. Macrophages were pre-incubated with GRC-ON89A-Hex for 12 h and then incubated with LPS (1 µg/mL) for 20 min. One-way analysis of variance (ANOVA) was used for comparison of group means, followed by Duncan *t*-test. Different letters indicated that the differences between the groups are significant at *p* < 0.05. Data are expressed as means ± SD of independent experiments (*n* ≥ 3).

**Figure 5:**
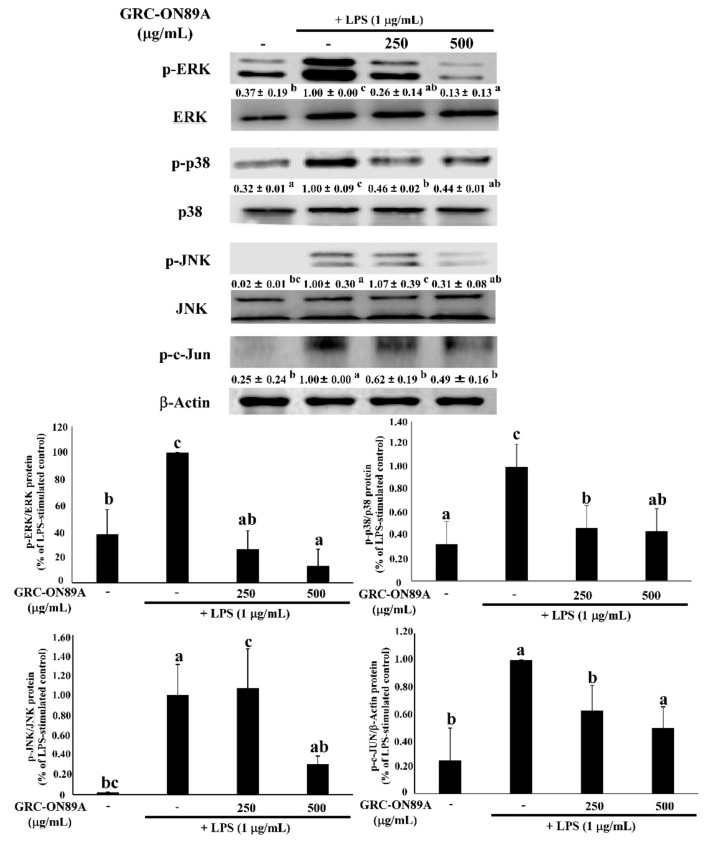
GRC-ON89A-Hex suppresses the phosphorylation of mitogen-activated protein kinase (MAPKs) in LPS-stimulated RAW264.7 macrophages. Macrophages were pre-incubated with GRC-ON89A-Hex for 12 h and then incubated with LPS (1 µg/mL) for 20 min. One-way analysis of variance (ANOVA) was used for comparison of group means, followed by Duncan *t*-test. Different letters indicated that the differences between the groups are significant at *p* < 0.05. Data are expressed as means ± SD of independent experiments (*n* ≥ 3). ERK, extracellular signal-regulated kinase; JNK, c-Jun N-terminal kinase.

Previous studies have reported that the transcription factor nuclear factor (NF)-κB is an important transcription factor modulating the expression of iNOS, COX2, and pro-inflammatory cytokines. Therefore, we checked whether GRC-ON89A-Hex blocked the NF-κB signaling pathway. Many studies were reported that inflammation is associated with the NF-κB and mitogen-activated protein kinases (MAPKs) signaling pathway [19]. In LPS-stimulated macrophages, pre-treatment with GRC-ON89A-Hex decreased the protein levels of phosphorylation of NF-κB p65 and IκBα (Figure 4). We also investigated the effects of GRC-ON89A-Hex on activation of MAPKs, extracellular signal-regulated kinase (ERK) 1/2, c-Jun N-terminal kinase (JNK), and p38 in the LPS-stimulated RAW 264.7 macrophages using western blot assay. LPS treatment increased phosphorylation of MAPKs, whereas pre-treatment with GRC-ON89A-Hex significantly suppressed their levels in LPS-stimulated RAW 264.7 macrophages (Figure 5). In addition, pre-treatment with GRC-ON89A-Hex suppressed the activation of the transcription factor c-Jun.

### 2.6. GRC-ON89A Reduces DNFB-Induced ACD in BALB/c Mice

**Figure 6:**
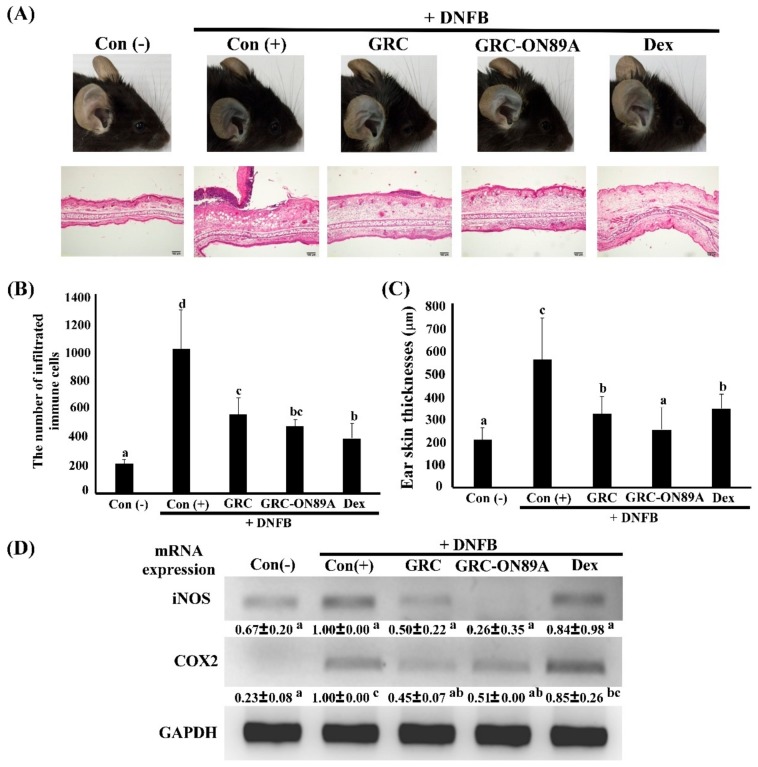
Effect of *Cordyceps militaris* grown on germinated *Rhynchosia nulubilis* (GRC) fermented with *Pediococcus pentosaceus* ON89A isolated from onion (GRC-ON89A) extracts on ear swelling and histopathological changes in 1-fluoro-2, 4-dinitrofluorobenzene (DNFB)-induced contact dermatitis in mice. (**A**) Representative images and histopathological analysis of GRC-ON89A treatment in DNFB-induced allergic contact dermatitis (ACD) in BALB/c mice. Extravasated Evans blue dye was observed in ears of mice. Ear tissue was stained using hematoxylin and eosin. Scale bars = 100 µm. (**B**) The number of infiltrated immune cells and (**C**) ear thickness of right ear were analyzed using a microscope with Metamorph software (Universal Imaging, West Chester, PA, USA). Data are expressed as means ± standard error (SE) of independent experiments (*n* ≥ 3, *p* < 0.05). (**D**) Effects of GRC-ON89A on iNOS and COX-2 mRNA expression in DNFB-induced contact dermatitis in ear of mice. Data are expressed as means ± SD of independent experiments (*n* ≥ 3, *p* < 0.05). One-way analysis of variance (ANOVA) was used for comparison of group means, followed by Duncan *t*-test. Different letters indicated that the differences between the groups are significant at *p* < 0.05.

Oral administration of GRC-ON89A (25 mg/kg) decreased the ear swelling of the mouse model (Figure 6A). In addition, the extravasation of Evans blue dye into the ear tissue was visually observed to decrease (Figure 6A). Histopathological analysis also demonstrated that DNFB-induced infiltration of immune cells into the ear tissue was suppressed by GRC-ON89A treatment (Figure 6B) and the DNFB-induced ear thickness was significantly reduced (Figure 6A,C).

## 3. Discussion

*C. militaris* has been used to treat numerous immune-related diseases, including ACD [20,21]. Previously, we reported that GRC-ON89A enhanced innate immunity, compared to GRC. Our study indicates that active compounds including adenosine, cordycepin, and polyphenol compounds were significantly increased in GRC after lactic acid bacteria fermentation. Adenosine inhibits inflammation by inhibiting Th17 differentiation and stimulating T-regulatory cell (Treg) differentiation of lymphocytes [22], inhibiting the production of NO and pro-inflammatory cytokines such as TNF-α and IL-1β [23,24,25,26] in macrophages and activated neutrophil adhesion to the vascular endothelium [25,26]. Polyphenols are the major compounds that act as primary antioxidants [27]. Reactive oxygen species and associated free radicals are involved in the development of human diseases containing inflammation and metabolic disorders [28]. We assumed that GRC-ON89A would exhibit enhanced anti-inflammatory activity and, therefore, we investigated its effect against LPS-induced inflammation and DNFB-induced hypersensitivity.

Inflammation is a central feature of many pathological conditions and is mediated by numerous cells including macrophages and lymphocytes [29]. Among them, macrophages are the major cells involved in the inflammatory processes. Activated macrophages overexpress NO, iNOS, and COX-2, and pro-inflammatory cytokines such as TNF-α and IL-1β [29,30]. iNOS is closely associated with pathological inflammation since it upregulates the production of NO that interacts with O^2−^ to form ONOO^−^, resulting in endothelial dysfunction and inflammatory responses [31]. COX2 produces prostaglandin E2 (PGE2) in activated macrophages from DNFB-sensitized mice [32]. PGE2 is the most abundant proteinoid, which affects inflammatory and immune events [33]. In this study, we observed that GRC-ON89A-Hex inhibited NO production by suppressing the level of iNOS mRNAs and proteins and decreased COX2 mRNAs and proteins (Figure 1). Activated macrophages also overproduce pro-inflammatory cytokines such as TNF-α and IL-1β [30]. Some reports suggested that production of these cytokines is required to produce NO and PGE2 in activated macrophages [18,34,35]. We observed that GRC-ON89A-Hex inhibited the expression of pro-inflammatory mediators in LPS-induced RAW 264.7 macrophages (Figure 3). Thus, anti-inflammatory activity of GRC-ON89A might be the result of the suppressive effects on the production of pro-inflammatory mediators and cytokines.

To understand the anti-inflammatory mechanisms of GRC-ON89A-Hex, we evaluated the protein expression levels of inhibitor of NF-κB, and IκBα. NF-κB is a central transcription factor that produces pro-inflammatory cytokines (e.g., TNF-α, IL-1β, IL-6, IL-8, and IL-12) that are closely related to ACD [36]. NF-κB exists mainly as a heterodimer consisting of subunits of p50 and p65 in an inactive cytoplasmic complex [37,38]. In the inactive state, NF-κB is associated with an inhibitory protein, IκBα. When NF-κB is stimulated by LPS, IκBα is phosphorylated and degraded, inducing NF-κB to translocate from the cytoplasm to the nucleus of macrophages [39,40]. The activation of NF-κB is triggered by MAPKs, which play an important regulatory role in both innate and adaptive immune systems responses [18,41]. Inflammatory inducers, including LPS, induce overproduction of cytokines by activating intracellular signaling pathways including MAPKs (ERK1/2, JNK, and p38) pathways in macrophages [18]. It has also been reported that JNK binds the NH2-terminal activation domain of c-Jun [42]. We observed that GRC-ON89A-Hex inhibited the expression of p-MAPKs and p-c-Jun. Therefore, these data suggest that GRC-ON89A contributed to attenuating inflammation by inhibiting NF-κB, MAPKs, and c-Jun. There are several reports that cordycepin and adenosine inhibited the activation NF-κB, MAPKs, and c-Jun [18,26,43]. It is reported that cordycepin inhibits the production of NO and COX-2 expression by suppressing MAPK signaling pathway, including p38, ERK, and JNK [18]. Adenosine is shown to inhibit proinflammatory outcomes by suppressing proinflammatory cytokine production and augmenting the anti-inflammatory cytokine, IL-10, production through binding to adenosine receptor [26,43,44]. As shown in Table 1, the content of cordycepin and adenosine is higher in GRC-ON89A than in GRC, which is consistent with enhanced the anti-inflammatory activity of GRC-ON89A in vivo. Our results suggest that adenosine and cordycepin inhibit the inflammatory actions by regulating adenosine receptors and NF-κB/MAPK signaling pathway.

Next, we carried out an in vivo ACD experiment. Repeated treatment with DNFB causes a skin contact hypersensitivity response such as an increase in ear swelling, and in vascular permeability [45]. The histological analysis data revealed that oral administration of GRC-ON89A suppressed DNFB-induced ear swelling and infiltration of immune cells into the dermis and epidermis. According to the previously reported studies, histopathologic examination of ACD and atopic dermatitis occurred hyperkeratosis, and increased numbers of lymphocytes, macrophages, and mast cells in dermis [46,47]. The histopathological images showed that GRC-ON89A reduced the number of infiltrated immune cells in DNFB-induced ACD mice. In addition, it is reported that DNFB-induced skin inflammation is caused by elevated levels of COX-2, iNOS, and several other proinflammatory mediators [48,49,50]. Enhanced anti-inflammatory activity of GRC-ON89A might be due to increased adenosine and cordycepin contents, which are known to inhibit the production of NO and pro-inflammatory cytokines such as iNOS, and COX-2 [18,51].

In summary, GRC-ON89A attenuated ACD symptoms and was more effective against ACD than GRC, increasing the yield of bioactive compounds such as adenosine and cordycepin. Further studies need to focus on identifying the novel bioactive compounds in GRG-ON89A and its underlying mechanism.

## 4. Materials and Methods

### 4.1. Preparation of GRC Fermented with Probiotic Strains

The GRC was prepared using patented technologies developed by Cell Activation Research Institution (CARI, Seoul, Korea), where a voucher specimen of the plant material was deposited (Kucari: 0903). *P. pentosaceus ON89A* were isolated from onion. *P. pentosaceus ON89A* isolated from onion strains used in this study was obtained from Dr. Y.-S. Park. GRC (5% *w*/*v*) was extracted with distilled water at 105 °C for 2 h and then inoculated with *P. pentosaceus ON89A* strains. GRC inoculated probiotic strain ON89A were heat-killed at 100 °C for 10 min and sonicated for 3 min (Sonics & Materials, Inc., Newtown, CT, USA) [14].

### 4.2. Preparation of GRC-ON89A-Hex

**Figure 7:**
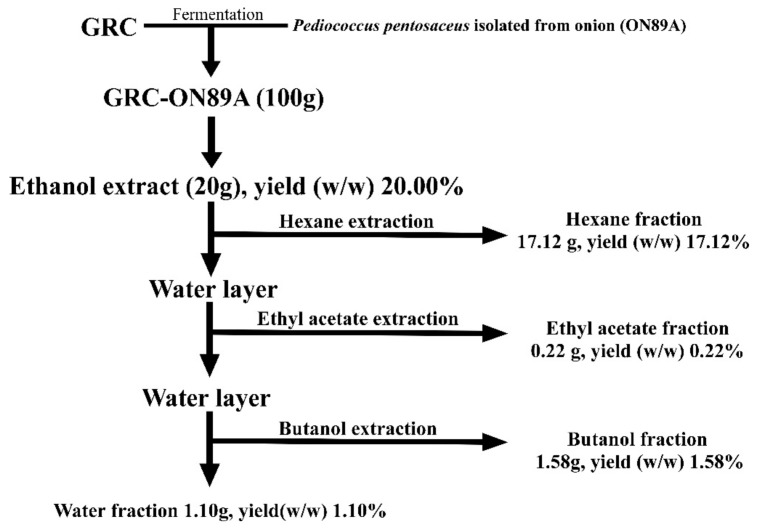
Fractionation of *Cordyceps militaris* grown on germinated *Rhynchosia nulubilis* (GRC) fermented with *Pediococcus pentosaceus* ON89A isolated from onion (GRC-ON89A).

GRC-ON89A (100 g) was solid-phase fermented after extraction for 72 h at 24–27 °C with 80% ethanol. After filtering, the ethanol extract was concentrated using a rotary evaporator, 20 g was dissolved in distilled water (200 mL) for 1 day, and then fractionated with n-hexane. The upper layer was concentrated using a rotary evaporator and the extract was obtained at 17.12 g (17.12% *w*/*w*). Similarly, ethyl acetate and n-butanol were used for fractionation based on polarity, and the fractions were obtained at 0.22 g (0.22%) and 1.58 g (1.58%), respectively. The fractionations were carried out at room temperature (Figure 7).

### 4.3. Quantitative Analysis of GRC and GRC-ON89A Extracts Using GC-TOF MS

Adenosine and cordycepin standards were purchased from Sigma-Aldrich (St. Louis, MO, USA). The freeze-dried samples (GRC, and GRC-ON89A) were reconstituted with 1 mL of the solvents initially used, aliquoted to a concentration of 500 μg/mL, and concentrated to complete dryness using a speed vacuum concentrator (Labogne ApS, Lynge, Denmark). Then, to protect ketone and aldehyde groups, 5 μL of 40 mg/mL methoxyamine hydrochloride (Sigma-Aldrich) in pyridine (Thermo-Fisher Scientific, Waltham, MA, USA) was added and shaken at 30 °C for 90 min. Then, 45 μL of *N*-methyl-*N*-trimethylsilyltrifluoroacetamide (MSTFA with 1% TMCS, Thermo) was added for trimethylsilylation and then incubated at 37 °C for 30 min [52].

A 0.5 μL aliquot of derivatized mixture was injected into the GC system (Agilent 7693 ALS, Agilent Technologies, Wilmington, DE, USA) in splitless mode with an Agilent 7890B gas chromatograph (Agilent Technologies, Wilmington, DE, USA) for chromatographic separation. Furthermore, we used a 30-m long, 0.25 mm i.d. Rtx-5Sil MS column with 0.25 m 95% dimethyl, 5% diphenyl polysiloxane film, and an additional 10-m integrated guard column (Restek, Bellefonte, PA, USA). The column temperature was held constant at 50 °C for 1 min, then increased at 20 °C/min to 330 °C, and kept at that temperature for 5 min [53].

The MS analysis was conducted using a LECO Pegasus HT TOF mass spectrometer controlled by the LECO ChromaTOF software 4.50 version (LECO, St. Joseph, MI, USA). The transfer line and ion source temperature were set to 280 °C and 250 °C, respectively. The mass spectra were recorded from 85 to 500 *m*/*z* at an acquisition rate of 17 spectra/s and 1800 V detector voltage. Data pre-processing was conducted using ChromaTOF software following acquisition of the apex mass values, entire spectrum, retention time, peak purity, and signal-to-noise ratio data [54].

### 4.4. Determination of TPC and TFC

The TPC of GRC and GRC-ON89A was measured according to a method described previously [55]. Briefly, the reaction mixture consisted of 50 µL of sample extracts, 100 µL Folin-Ciocalteu regent, and 750 µL 7% sodium carbonate solution, and gallic acid was used as the standard. The absorbance was read at 720 nm after a 30 min reaction at an ambient temperature. The results were reported as milligram GAE per gram (mg GAE/g) of GRC and GRC-ON89A. The TFC of GRC and GRC-ON89A was determined using a method described previously [55]. In brief, 50 µL each of GRC and GRC-ON89A was mixed with 15 µL 5% sodium nitrite solution, 15 µL 10% aluminum nitrate solution was added after 5 min, and then 100 µL 1 N sodium hydroxide solution was added to the mixture. The absorbance was read at 510 nm after 11 min reaction at an ambient temperature in a dark room. Quercetin was used as the standard and the results were reported as milligram QE per gram (mg QE/g) of GRC and GRC-ON89A.

### 4.5. Cell Culture

RAW 264.7 macrophages were purchased from the Korean Cell Line Bank (KCLB, Seoul, Korea). They were cultured in Dulbecco’s modified Eagle’s medium (Welgene, Seoul, Korea) supplemented with 10% fetal bovine serum (Welgene) and 100 U/mL penicillin and streptomycin (Welgene). The cells were grown in a 75 cm^2^ culture flask at 37.5 °C in an atmosphere of 5% CO_2_ under humidified atmospheric pressure.

### 4.6. RAW 264.7 Macrophages Viability

RAW 264.7 macrophages were measured using the Cell Counting Kit-8 (CCK-8) assay (DOJINDO Laboratories, Kumamoto, Japan), as described previously [56]. The cells (2 × 10^4^ cells/well) were plated onto a 96 well plate and treated with various concentrations (250, 500, and 1000 μg/mL) of GRC-ON89A-Hex for 48 h. The CCK-8 solution was added, and cells were incubated for 2 h. After adding CCK-8 solutions, the percentage cell viability was measured using a microplate reader (Epoch, Biotek Instruments, Inc., Winooski, VT, USA) at 450 nm.

### 4.7. Measurement of NO Production

NO production by RAW 264.7 macrophages was measured in the culture medium using the Griess reaction as described previously [57] using nitrite concentration as an indicator of NO production. RAW 264.7 macrophages (2 × 10^4^ cells/well) were treated with GRC-ON89A-Hex (250, 500, and 1000 μg/mL) for 1 h and then stimulated with LPS for 24 h. NO production was measured using Griess reagent (1% sulfanilamide, 0.1% naphthylenediamine dihydrochloride, and 0.5% H_3_PO_4_). The absorbance was measured using a microplate reader (Epoch, Biotek Instruments, Inc., Winooski, VT, USA) at 450 nm.

### 4.8. RNA Isolation and RT-PCR

RT-PCR was performed for the detection of the mRNA expression of iNOS, COX-2, IL-1β, and TNF-α, with glyceraldehyde 3-phosphate dehydrogenase (GAPDH) as the internal, housekeeping control gene. After stimulating the RAW 264.7 macrophages with LPS (1 µg/mL) for 6 h, total cellular RNA was isolated using TRIzol™ reagent (Life Technologies, Carlsbad, CA, USA) according to the manufacturer’s instructions. In addition, to evaluate the effect of GRC-ON89A on the ear of DNFB-induced mice, the level of COX-2 and iNOS mRNA expression were measured by RT-PCR analysis. The ear tissues were homogenized by Handheld Homogenizer (Hangzhou Miu Instruments Co., Ltd., Hangzhou, China). Subsequently, an RT reaction was carried out using a kit to produce cDNA (First Strand cDNA synthesis kit, ThermoFisher Scientific, Waltham, MA, USA). Reverse transcriptase PCR was conducted according to the manufacturer’s instructions (Cosmogenetech Co., Seoul, Korea). The following conditions were used for each PCR reaction: 94 °C for 2 min (1 cycle); 94 °C for 20 s, 60 °C for 10 s, and 72 °C for 30 s (35 cycles); and a final extension phase at 72 °C for 5 min. The primer sequences for the analysis of mRNA expression were as follows: iNOS, 5′-CTT CAA CAC CAA GGT TGT CTG CA-3′ (forward) and 5′-ATG TCA TGA GCA AAG GCG CAG AA-3′ (reverse); COX-2, 5′-AAC CGT GGG GAA TGT ATG AGC A-3′ (forward) and 5′-AAC TCT CTC CGT AGA ACC TTT TCC A-3′ (reverse); IL-1β, 5′-TAC AAG GAG AAC CAA GCA ACG ACA-3′ (forward) and 5′-TGT CGT TGC TTG GTT CTC CTT GTA-3′ (reverse); TNF-α, 5′-ATG AGC ACA GAA AGC ATG ATC CG-3′ (forward) and 5′-CCA AAG TAG ACC TGC CCG GAC TC-3′ (reverse); and GAPDH, 5′-CAT ATT TCT CGT GGT TCA CAC CC-3′ (forward) and 5′-CAT ATT TCT CGT GGT TCA CAC CC-3′ (reverse). The PCR products were electrophoresed on 1.5% agarose gels (iNtRON Biotechnology, Seongnam, Korea). The bands were scanned and analyzed using chemiluminescence with the Odyssey LCI Image software (LI-COR Biosciences, Lincoln, NE, USA).

### 4.9. Western Blot Analysis

The RAW 264.7 macrophages were incubated with various concentrations of GRC-ON89A-Hex for 2 h prior to LPS treatment. Cells were washed with phosphate-buffered saline (PBS) and lysed in radioimmunoprecipitation assay (RIPA) lysis buffer (iNtRON Biotechnology, Seongnam, Korea). Protein concentrations were determined using the Pierce bicinchoninic acid (BCA) protein assay kit (ThermoFisher Scientific). Equal amounts of protein were separated using 10% sodium dodecyl sulfate polyacrylamide gel electrophoresis. The proteins were transferred to nitrocellulose membranes (Bio-Rad Laboratories, Inc., Hercules, CA, USA) and blocked in 5% skim milk in Tris-buffered saline plus Tween (TBST, 20 mM Tris, 500 mM sodium chloride (pH 7.6), and 0.1% Tween 20) for 1 h at room temperature. Specific antibodies against inducible iNOS (1:1000, Santa Cruz, CA, USA), COX-2, NF-κB p65, p-NF-κB p65, IκBα, p-IκBα, ERK, p-ERK 1/2, JNK, p-JNK, p38, p-p38, c-Jun N terminal kinase, p-c-Jun (1:1000; Cell signaling Technology, Danvers, MA, USA), and β-Actin (1:2000; Santa Cruz, CA, USA). Then, the membranes were washed thoroughly with TBST, followed by incubation with horseradish peroxidase-conjugated secondary antibodies (Santa Cruz) for 2 h. The blots were detected using an enhanced chemiluminescence western blotting detection system with the Odyssey LCI Image software (LI-COR Biosciences, Lincoln, NE, USA).

### 4.10. Experimental Design

The 7-week-old mice used in this study were obtained from Orient Bio (Eumsung, Korea) and maintained as described previously [14]. Experiments were performed in accordance with the Institutional Animal Care and Use Committee (IACUC) guidelines (GIACUC-R2018001) at Gachon University (Gyeonggi-do, Korea). The dorsal skin of each mouse was shaved and sensitized with 100 µL 0.5% DNFB in acetone/olive oil (4:1, *v*/*v*) for 1, 2, 3, 5, and 7 days. The mice were topically treated with 25 µL 0.2% DNFB, which was applied to the surface of the right ears for 9 days. The control group was treated with acetone/olive oil (4:1, *v*/*v*). Dexamethasone (3 mg/kg) treatment was used as a reference drug and 25 mg/kg of GRC-ON89A was orally administered for 10 days. Con (-) group and non-DNFB-treated group is orally administered D.W.

### 4.11. Histopathological Examination of Animal Tissue

The histopathological analysis was performed using previously described methods [2,14]. The ear tissue was embedded in paraffin blocks and sectioned at 5 µm thickness, and the tissue sections were stained with H&E stain solution. Images were acquired from representative section using a Nikon Eclipse Ti microscope (Point Grey Research, Richmond, BC, Canada) at a fixed magnification of 40×.

### 4.12. Statistical Analysis

The data are expressed as the means ± standard deviation (SD) or standard error (SE) and were analyzed using a one-way analysis of variance (ANOVA) Duncan *t*-test using the statistical package for the social sciences (SPSS) software, version 12 (SPSS Inc., Chicago, IL, USA). Different letters indicated that the differences between the groups are significant at *p* < 0.05.

## Figures and Tables

**Table 1:** Cordycepin and adenosine content in *Cordyceps militaris* grown on germinated *Rhynchosia nulubilis* (GRC) and GRC fermented with *Pediococcus pentosaceus* ON89A isolated from onion (GRC-ON89A).

Target Bioactive Compounds	GRC-ON89A	GRC
Adenosine (mg/g)	7.03 ± 0.15	3.88 ± 0.08
Cordycepin (μg/g)	1053.33 ± 11.81	180.58 ± 1.54

Data are expressed as means ± standard error (SE).

**Table 2:** Total polyphenol and flavonoid content in *Cordyceps militaris* grown on germinated *Rhynchosia nulubilis* (GRC) and GRC fermented with *Pediococcus pentosaceus* ON89A isolated from onion (GRC-ON89A).

Compounds	GRC-ON89A	GRC
Total polyphenol contents (mg GAE/g)	15.92 ± 1.20 ***	7.65 ± 1.70
Total flavonoid contents (mg QE/g)	7.47 ± 0.27 ***	5.37 ± 0.48

GAE: gallic acid equivalent; QE, quercetin equivalent. Data are expressed as means ± standard deviation (SD, *** *p* < 0.005 vs. GRC).
